# Repetitive Transcranial Magnetic Stimulation Does Not Improve the Sequence Effect in Freezing of Gait

**DOI:** 10.1155/2019/2196195

**Published:** 2019-06-04

**Authors:** Jinghong Ma, Linlin Gao, Taomian Mi, Junyan Sun, Piu Chan, Tao Wu

**Affiliations:** ^1^Department of Neurology, Xuanwu Hospital of Capital Medical University, Beijing, China; ^2^Department of Neurobiology, Key Laboratory on Neurodegenerative Disorders of Ministry of Education, Beijing Institute of Geriatrics, Xuanwu Hospital, Capital Medical University, Beijing, China; ^3^Beijing Key Laboratory on Parkinson's Disease, Parkinson Disease Center of Beijing Institute for Brain Disorders, Beijing, China; ^4^Department of Neurology, Neurobiology and Geriatrics, Xuanwu Hospital of Capital Medical University, Beijing Institute of Brain Disorders, Beijing, China; ^5^Clinical Center for Parkinson's Disease, Capital Medical University, Beijing, China; ^6^National Clinical Research Center for Geriatric Disorders, Beijing, China

## Abstract

**Introduction:**

The sequence effect (SE) is a reason contributing to freezing of gait (FOG) in Parkinson's disease (PD) patients. There is no effective treatment for the SE. The objective of the current study is to investigate the effect of repetitive transcranial magnetic stimulation (rTMS) on the SE in PD patients with FOG.

**Methods:**

28 PD patients with FOG received either real or sham 10-Hz rTMS over the supplementary motor area (SMA). The effects of rTMS on the SE, FOG, and some gait parameters were evaluated.

**Results:**

rTMS did not improve the SE. Real rTMS had beneficial effects on FOG and some gait parameters, and this effect lasted for at least four weeks.

**Conclusions:**

High-frequency rTMS over the SMA cannot alleviate the SE in PD patients with FOG. rTMS has a long-lasting beneficial effect on FOG; however, this effect is not achieved by improving the SE but may be through improving some other gait parameters.

## 1. Introduction

Freezing of gait (FOG) is a disabling and common symptom in Parkinson's disease (PD) characterized by brief episodes of inability to step or by extremely short steps that typically occur on initiating gait or on turning while walking [1, 2]. The mechanisms underlying FOG are poorly understood. Impairments in rhythmicity [3], symmetry [4], and bilateral coordination [5] have been reported to be associated with FOG episodes. In addition, diminished stride length is also a critical factor that results in FOG [6]. Nieuwboer et al. [7] suggested that freezing whilst walking could stem from stride-to-stride variability, which results in failure to generate normal amplitude in step length, comparing with those that do not experience freezing [8, 9]. This magnitude of stride-to-stride fluctuations further increase in patients in the “off” state [3, 8, 10], hastening, or an increase in cadence with a decrease in step length, often deteriorate FOG [7]. In PD patients, the decreased amplitudes might further destabilize normal gaits and induce a vicious circle of progressively shorter step length, resulting in FOG [7]. This progressive decrease in amplitude of sequential movements is called the sequence effect (SE), which is a common feature in PD patients [10].

The treatment of FOG is difficult. As the SE has been suggested as a reason contributing to FOG [10, 11], alleviating the SE should be an approach to help improve FOG. However, it has been demonstrated that levodopa has no impact on the SE [10]. Therefore, development of new effective therapeutic strategies is necessary. Repetitive transcranial magnetic stimulation (rTMS) is a noninvasive method to stimulate the human brain, and high-frequency facilitatory rTMS has been shown improving motor symptoms in PD patients. Despite the discrepant results [12–15], there are increasing studies that have reported benefit effects of rTMS on FOG [16]. A previous study found that rTMS has no effect on the SE during hand movement [17]. However, whether rTMS could alleviate the SE in FOG has never been investigated. We thus investigated the potential benefits of rTMS on the SE in PD patients with FOG in the current study.

## 2. Methods

### 2.1. Participants

PD patients were diagnosed according to the MDS Clinical Diagnostic Criteria and were recruited from the Movement Disorders Clinic of the Xuanwu Hospital of Capital Medical University. 30 idiopathic PD patients with FOG were identified using the item 3 of the FOG questionnaire (a positive answer to FOG-Q3—“Do you feel as if your feet are glued to the floor while walking, making a turn, or while trying to initiate walking?”). In 24 of the 30 (80%) self-reported freezers, FOG was recorded during clinical testing or spontaneous behavior. Subjects were included if they were able to walk 10 meters repeatedly more than 3 times without aids. Patients with other neurological or orthopedic conditions that might affect gait or posture, comorbidities of neurological disease other than PD, history of deep brain stimulation surgery, or MMSE score ≤24 were excluded. 2 participants were excluded because of deficit of cognitive ability. The experiments were performed according to the Declaration of Helsinki and were approved by the Institutional Review Board of Xuanwu Hospital. The rTMS study was registered at the Clinical Trial Registration (URL: http://www.clinicaltrials.gov.), unique identifier: NCT03219892. Written informed consent was obtained from all participants prior to the experiment.

At least after a 12-hour withdrawal of anti-Parkinson medication, clinical assessments of patients were conducted in their practical off state, including the Movement Disorder Society-Sponsored Revision Unified Parkinson's Disease Rating Scale (MDS-UPDRS), Hoehn and Yahr (H&Y) stage, Montreal Cognitive Assessment (MoCA) Beijing version, Mini-Mental State Examination (MMSE), Hamilton Anxiety Scale (HAMA), Hamilton Depression Scale (HAMD) 17, FOGQ, and parts II and III of the NFOGQ [18] (Supplementary Table 1).

### 2.2. Gait Assessments

To measure the spatial and temporal gait parameters, an electronic walkway GAITRite (CIR Systems Inc. Clifton, NJ 07012) was employed. Measuring 5.2 m long and 0.89 m wide, the GAITRite collects data through pressure sensors embedded into the carpet. The GAITRite has been found to produce highly reliable measurements, particularly with walking speed, cadence, and step length (intraclass correlations between 0.82 and 0.92 and coefficients of variation between 1.4% and 3.5%) [19]. The GAITRite was positioned in an open space of the center of an outpatient hall so that there was at least 3 meters of space on each side. This arrangement provided sufficient open space to minimize environmental stimuli that may have provoked freezing [20].

Gait assessments were performed in the on state. Participants were instructed to stand still at the starting point of the carpet, walked at the middle rather than the bilateral margin of the carpet, and stopped at the end of the carpet in the on state. All participants walked barefoot along the mat 3 times in a usual speed. When calculating the regression slopes of walking trials, step length for each footstep was measured, while the first and last steps were excluded to avoid patients' instability and limitation of the carpet. Spatiotemporal data for each trial were identified from the second strides within the capture zone, after gait initiation at the beginning of the data capture area. The ambulation time, mean velocity, step count, and mean cadence were measured. The values measured in the 3 walking trails were averaged in each subject. The step length was plotted against step number in each walking trial. Linear regression was used to determine the slope of each regression curve. The averaged regression slope (*b*) for the 3 walk trails was used to represent the SE in each participant [21]. Once freezing episodes did occur during the walking, and we asked the patients to stop and have a rest. The experiment was repeated when the patients were in a better state until we collected adequate data.

### 2.3. rTMS Study

#### 2.3.1. Study Design

This experiment was a double-blind, placebo-controlled, single-center trial with a parallel design consisting of two parts: 10-Hz rTMS over the supplementary motor area (SMA, real group) and sham stimulation (sham group) at the practical “on” state. 28 patients were randomized about 2 : 1 into the two groups, to receive either real (*N*=18) or sham (*N*=10) rTMS protocol. High-frequency rTMS on the bilateral primary motor cortex [16, 22] or SMA [23] has been shown improving FOG in PD patients. A recent study found that rTMS in the SMA had more benefit on FOG than stimulation in the motor cortex [24]. In addition, it has been shown that rTMS on the motor cortex did not improve the SE during hand movements [11]. Therefore, we chose the SMA as the stimulate target in the present study. One of the authors Junyan Sun determined the allocation and group, and it was concealed to both physicians and participants involved throughout the whole course of the study. Patients kept previous medication treatment throughout the trial. The intervention of rTMS was performed at the same time of day for each patient.

#### 2.3.2. Real and Sham rTMS Protocol

We performed the real or sham rTMS in ten sessions over two successive weeks, one session per day for five consecutive days per week. For the real rTMS, a 7-cm handheld figure-of-8 coil was connected to a biphasic magnetic stimulator (Magstim Rapid; Magstim Co. Ltd., UK). To apply focal rTMS over the SMA, the stimulation site was determined as the site 3 cm anterior to the leg motor area along with the midline [25]. The coil was held so that the induced current was perpendicular to the midline. The stimulus intensity was set at the 90% rest motor threshold for the right tibialis anterior muscle when the leg primary motor area was stimulated. In each session, a 5-second burst of 10-Hz rTMS was repeated 20 times at every minute (in total, 1,000 pulses and 20 minutes' duration). For the sham rTMS, the same stimulation parameters were used, but the coil was placed in 90° turning angulation over the SMA so that no relevant current flow was induced in the cortical tissue [26, 27].

#### 2.3.3. Clinical and Gait Assessments

**Figure 1:**
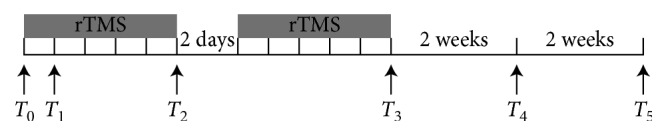
Flow of participants.

**Figure 2:**
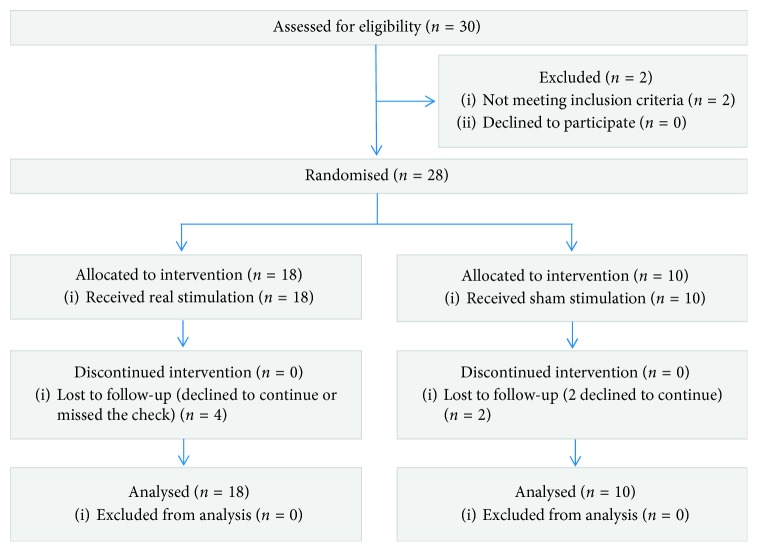


The assessments were carried out in the clinical “on” state at the same time of the day. Baseline and follow-up evaluations (including MDS-UPDRS III and gait assessment) for each participant were performed before rTMS (baseline) and after the 1st, 5th, 10th sessions and then 2 weeks and 4 weeks after the last session, defined as *T*_0_, *T*_1_, *T*_2_, *T*_3_, *T*_4_, and *T*_5_, respectively. In addition, FOG-Q was evaluated at *T*_0_, *T*_3_, and *T*_5_, respectively. The primary outcome was the rTMS effect on SE. We included FOG-Q as a secondary clinical outcome to evaluate the improvement of FOG. Additionally, MDS-UPDRS III and gait assessment (including ambulation time, cadence, step count, and velocity) is adopted. The flow of participants is presented in Figure 1, and the flow of the research is listed in Figure 2.

### 2.4. Statistics Analysis

Demographic data were presented as mean ± SD for continuous variables. An independent two samples *t*-test was performed for the comparison of continuous variables, and the chi-square test was used to compare categorical variables. We applied mixed effect model repeated measures (MMRM) by SPSS 22 to estimate the effect of rTMS on the sequence effect (the averaged regression slope (*b*) for the 3 walk trails), FOG-Q scores, MDS-UPDRS III scores, and other gait parameters. For each variable, we applied a separate model where the independent variables were the group (real rTMS and sham rTMS) and the visit (*T*_0_, *T*_1_, *T*_2_, *T*_3_, *T*_4_, and *T*_5_) and the group *∗* visit condition interaction term. The threshold for the level of significance was set at *α* = 0.05 (Bonferroni correction).

## 3. Results

### 3.1. Participants

Participant demographics and clinical features are described in Supplementary Table 1. There was no significant difference between the two groups in any clinical assessments. Seven patients in the real group had difficulty in initiation, while four patients in the sham group experienced this problem. There was no significant difference between the two groups on this phenomenon (Supplementary Table 1). In the real rTMS group, 2 patients missed the check at *T*_4_ and 2 patients dropped out at *T*_4_ and *T*_5_. In the sham group, 2 patients dropped out at *T*_4_ and *T*_5_. We filled the gaps with the group average. No adverse reactions to the rTMS were reported.

### 3.2. Sequence Effect

**Figure 3:**
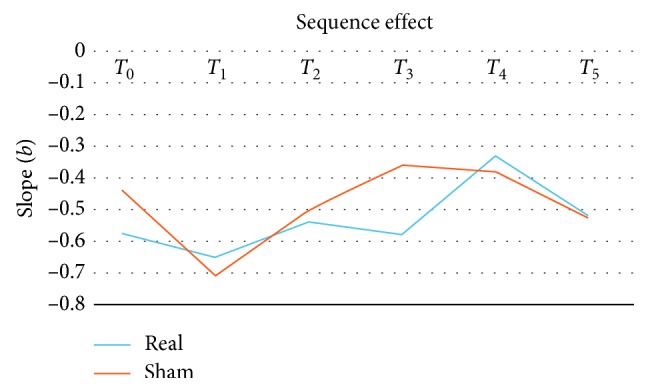
Mean change of the real and sham group with the time points.

There was no difference of the SE between the groups at the baseline. Analysis of regression slope (*b*) values did not show significant group *∗* visit interaction. Both real and sham rTMS had no effect on the SE (Table 1, MMRM, *p* > 0.05).  Figure 3 shows the mean change of the real and sham groups with the time points.

### 3.3. Clinical and Gait Assessments

**Figure 4:**
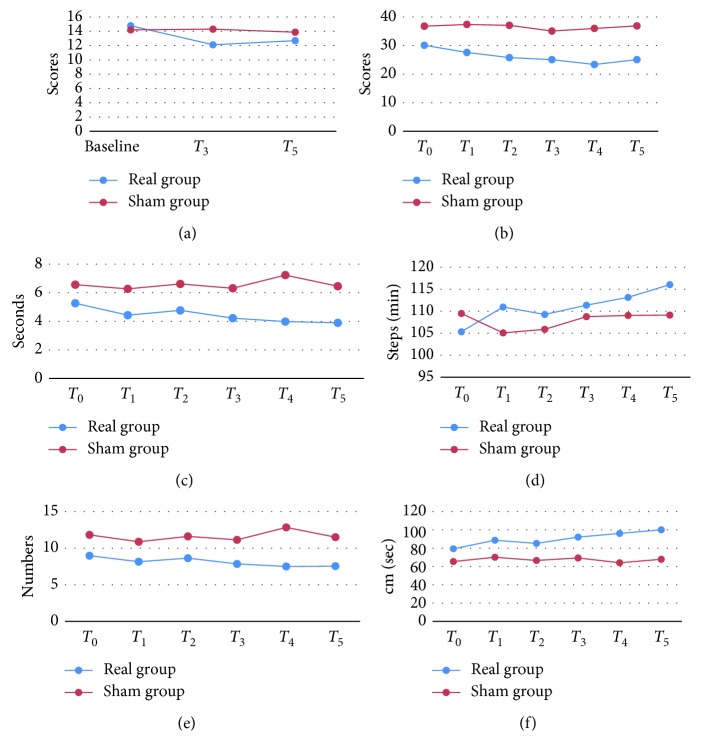
Change of each trail and examination. These results indicated that the real rTMS has an improved effect on (a) FOG-Q, (b) MDS-UPDRS III, (c) ambulation time, (d) cadence, (e) step count, and (f) velocity.

In the comparison of other measurements between the real and sham rTMS group, there was a significant interaction between group and visit in FOG-Q, ambulation time, cadence, step count, and velocity. Post hoc analysis showed significantly decreased FOG-Q, MDS-UPDRS III;, ambulation time, and step count, as well as increased cadence and velocity in the real group (Table 2), and the mean values are showed in Supplementary Table 2. We found that real rTMS significantly improved items 2 (facial expression) and 11 (freezing of gait) of MDS-UPDRS III; (Supplementary Table 3). In the real group, the FOG-Q was improved at the *T*_3_ and *T*_5_. The MDS-UPDRS III; scores were significantly decreased from *T*_3_ to *T*_5_ in the real group. Score changes from baseline at *T*_3_, *T*_4_ and *T*_5_ were −4.95 (*p*=0.002), −6.56 (*p* ≤ 0.001), and −4.95 (*p*=0.004), respectively. There were significant changes of ambulation time and cadence at *T*_5_ compared to the baseline and improvement of velocity at *T*_4_. These results indicated that the real rTMS has an improved effect on FOG-Q, MDS-UPDRS III;, ambulation time, cadence, step count, and velocity. No significant changes were found in the sham group.  Figure 4 shows the changes of these assessments across the study in both groups.

## 4. Discussion

The current research investigated the effect of rTMS on the SE in PD patients with FOG. Contrary to our expectation, high-frequency rTMS did not improve the SE. In contrast, we found that high-frequency rTMS focusing on the SMA can improve FOG, general motor symptoms, and gait performance. Our result together with previous finding indicates that the SE did not respond to levodopa treatment, approving there is still no effective treatment for the SE [10, 16, 17]. We need to develop new therapeutic strategies in future.

PD patients with FOG often have difficulty in initiating the walking sequence to begin with and have short, slow steps when they achieve steady-state walking or turning, and some patients can even freeze when take a small turn [1]. After they recommence walking (often with difficulty), further freezing episodes can occur according to environmental triggers, task constraints, and the ability of the person to compensate using cognitive strategies [28]. As focused on the SE, we only recorded the step length during the straight walking, but not during turning.

Cunnington et al. found that individuals with PD generated gradually slowing down movement comparing with controls [10, 16, 27, 29]. SE occurs in sequential automatic movement in the absence of external cues and without attention-focused motor control, such as walking [6, 10]. Nieuwboer et al. and colleagues found the phenomenon of SE in the last three steps preceding a freezing episode in PD patients [7, 30]. Although the SE is a common feature in PD [10, 31] and is a reason contributing to the FOG [10], our understanding on this problem remains limited. It has been speculated that the SE is induced by fatigue [31–33]. However, later investigations showed that fatigue is unlikely a critical reason underlying the SE [11]. A recent report has suggested that higher energetic cost may contribute to the SE [33]. Only few studies have investigated the neural mechanisms underlying the SE, and most of them focused on the SE in hand movement, such as progressive micrographia and gradually slow movement. Reduced motor cortex plasticity [13], functional disconnection between the SMA, rostral cingulate motor area, and cerebellum [11], or reduced volume in the anterior cingulate cortex and cerebellum [34] have been related to the SE. However, as rTMS targeting on either motor cortex [10] or SMA (the current study) has no impact on the SE, it is likely neural networks outside these motor circuits should be also involved in the genesis of the SE. As a clear understanding of neural correlates is critical in developing new therapeutic strategies of the SE, we need to put more efforts in this area.

Our results showed that 10 Hz rTMS over the SMA could significantly improve FOG-Q at *T*_3_ and *T*_5_, respectively, which indicates that rTMS could alleviate FOG in PD, and this effect lasted for at least four weeks after the end of the therapy. This finding is consistent with previous reports of benefit effects of rTMS on FOG [9–12]. We also found significant influence of rTMS on some gait parameters, including decreased ambulation time and step count, as well as increased cadence and velocity. A reduced step count reflects an increased stride length. As our measurement tool, “GAITRite” did not record the stride length for each trail directly, and we calculated averaged stride length in each time as the length of walking divided by the numbers of step count (Supplementary Table 4). Although the impact of rTMS on the stride length did not achieve the significant level (post hoc analysis), there was a trend of increasing stride length in the real group. Diminished stride length and step velocity are associated with FOG in PD patients [35]. Our findings demonstrated that high-frequency rTMS could alleviate FOG by improving stride length and velocity. In addition, rTMS improved MDS-UPDRS III scores, which indicate that high-frequency rTMS could improve general motor symptoms in PD. These findings together approve that high-frequency rTMS could alleviate FOG in PD patients; however, this effect is not achieved by improving the SE but may be through improving some other gait performances.

It has been approved that attention could improve gait problems (e.g., diminished stride length), as PD patients can use attentional control to bypass impaired automatic control to maintain movements [6, 35]. However, as we have asked the patients try to keep the same condition in each gait evaluation, moreover, the patients who received sham stimulation did not show significant change of stride length; the improvement of stride length was mainly a result of rTMS treatment. Attention unlikely had significant impact on our results.

There are some limitations in this study. First, to avoid the falls, the patients were investigated in their on state. Our results can only reveal the effect of rTMS as an add-on therapy. Second, due to the small sample size, we did not divide the patients with FOG into subgroups according to their phenotypes (e.g., freezing while initiating, freezing while turning, and freezing while straightly walking). More patients should be recruited in future study. Third, we did not use the TMS navigation system to localize the SMA, which will be improved in future studies.

## 5. Conclusion

In summary, the present study shows that high-frequency rTMS over the SMA cannot alleviate the SE in PD patients with FOG. In contrast, rTMS has a long-lasting beneficial effect on FOG, which is not achieved by alleviating the SE, but may be by improving other gait performances.

## Figures and Tables

**Table 1:** Comparison of the sequence effect between and within the groups.

*T*_*n*_	Real group (mean ± SD)	Sham group (mean ± SD)	MMRM	*p* value	Post hoc *p* value	
*T*_0_	−0.611 ± 0.319	−0.521 ± 0.422	Group	0.782	Real	Sham
*T*_1_	−0.718 ± 0.446	−0.797 ± 0.591	Visit	0.287	1.000	1.000
*T*_2_	−0.539 ± 0.670	−0.508 ± 0.397	Group *∗* visit	0.641	1.000	1.000
*T*_3_	−0.744 ± 0.820	−0.385 ± 0.185			1.000	1.000
*T*_4_	−0.281 ± 0.731	−0.430 ± 0.348			1.000	1.000
*T*_5_	−0.644 ± 0.531	−0.612 ± 0.267			1.000	1.000

Post hoc: comparing with *T*_0_; *T*_*n*_: time points; SD: standard deviation; MMRM: mixed effect model repeated measures.

**Table 2:** Changes of clinical and gait assessments across the study.

MMRM	DF	*F* value	*p* value	*T*_*n*_	Post hoc (*p* value)	
					Real group	Sham group
*FOG-Q*						
Group	1	0.280	0.601	*T*_3_	0.003^*∗*^	1.000
Visit	2	3.641	0.033^*∗*^	*T*_5_	0.023^*∗*^	1.000
Group *∗* visit	2	3.445	0.039^*∗*^			
*MDS-UPDRS III*						
Group	1	0.941	0.341	*T*_1_	1.000	1.000
Visit	5	3.576	0.005^*∗*^	*T*_2_	0.038^*∗*^	1.000
Group *∗* visit	5	1.158	0.334	*T*_3_	0.002^*∗*^	1.000
				*T*_4_	0.000^*∗*^	1.000
				*T*_5_	0.004^*∗*^	1.000
*Ambulation time (seconds)*						
Group	1	8.535	0.007^*∗*^	*T*_1_	0.048^*∗*^	1.000
Visit	5	2.919	0.016^*∗*^	*T*_2_	1.000	1.000
Group *∗* visit	5	3.158	0.010^*∗*^	*T*_3_	0.004^*∗*^	1.000
				*T*_4_	0.000^*∗*^	1.000
				*T*_5_	0.000^*∗*^	1.000
*Cadence (steps/min)*						
Group	1	0.721	0.404	*T*_1_	0.241	1.000
Visit	5	3.214	0.009^*∗*^	*T*_2_	1.000	1.000
Group *∗* visit	5	2.788	0.020^*∗*^	*T*_3_	0.178	1.000
				*T*_4_	0.021^*∗*^	1.000
				*T*_5_	0.000^*∗*^	1.000
*Step count*						
Group	1	7.834	0.010^*∗*^	*T*_1_	0.871	1.000
Visit	5	2.008	0.082	*T*_2_	1.000	1.000
Group *∗* visit	5	2.446	0.038^*∗*^	*T*_3_	0.090	1.000
				*T*_4_	0.007^*∗*^	1.000
				*T*_5_	0.009^*∗*^	1.000
*Velocity (cm/sec)*						
Group	1	6.471	0.018^*∗*^	*T*_1_	0.190	1.000
Visit	1	4.890	0.000^*∗*^	*T*_2_	1.000	1.000
Group *∗* visit	5	3.381	0.007^*∗*^	*T*_3_	0.010^*∗*^	1.000
				*T*_4_	0.000^*∗*^	1.000
				*T*_5_	0.000^*∗*^	1.000

*T*_*n*_: test number; post hoc: comparing with *T*_0_; MMRM: mixed effect model repeated measures; DF: degree of freedom. ^*∗*^ *p* < 0.05.

## Data Availability

Readers can access the data supporting the conclusions of the study from the supplementary information.

## Supplementary Materials

Supplementary Materials Supplementary Table 1: demographics and clinical features. Supplementary Table 2: values of clinical and gait assessments across the study. Supplementary Table 3: changes of MDS-UPDRS III across the study. Supplementary Table 4: values of cadence and stride length assessments across the study.
