# Supplementary material for: Repetitive Transcranial Magnetic Stimulation Does Not Improve the Sequence Effect in Freezing of Gait
Source: Parkinsons Dis. 2019 Jun 4;2019:2196195. doi: 10.1155/2019/2196195 (PMC6589230; doi:10.1155/2019/2196195)
Supplement: Supplementary Materials — Supplementary Table 1: demographics and clinical features. Supplementary Table 2: values of clinical and gait assessments across the study. Supplementary Table 3: changes of MDS-UPDRS III across the study. Supplementary Table 4: values of cadence and stride length assessments across the study. [file 2196195.f1.pdf]

Supplementary Table 1. Demographics and clinical features

	Real Group (Mean ±SD)	Sham Group (Mean ±SD)	P value
Sex (M/F)	18 (8/10)	10 (5/5)	0.080[c]
Age (years)	59.94 ±9.16	66.00 ±8.55	0.098[a]
Disease Duration (years)	8.94 ±5.48	7.50 ±4.72	0.490 [a]
FOG duration (years)	3.08 ±4.05	2.95 ±1.77	0.261[b]
LEDD (mg)	691.39 ±321.27	588.80 ±258.47	0.395 [a]
MDS-UPDRS III	38.11 ±16.20	41.10 ±19.72	0.669 [a]
H&Y	2.42 ±0.60	2.40 ±0.94	0.980[b]
FOGQ	14.78 ±5.59	14.20 ±3.43	0.770[a]
NFOGQ2	14.33 ±4.31	13.40 ±3.72	0.383[b]
NFOGQ3	5.50 ±2.79	5.80 ±1.93	0.865[b]
MMSE	28.67 ±2.14	29.10 ±1.60	0.582[b]
MOCA	26.50 ±4.59	24.80 ±4.34	0.383[b]
HAMD	7.28 ±4.52	6.60 ±3.06	0.885[b]
HAMA	9.89 ±7.16	12.20 ±8.98	0.461 [a]
Initiation difficulty(with/without)	7/11	4/6	0.954 [c]

a: Independent Two Sample T test; b: Rank sum test; c: chi-squared test

SD: Standard deviation

Supplementary Table 2 The values of clinical and gait assessments across the study.

	Real Group (Mean ±SD)	Sham Group (Mean ±SD)		Real Group (Mean ±SD)	Sham Group (Mean ±SD)
FOG-Q			MDS-UPDRS III		
T0	14.78±5.59	14.20±3.42	T0	30.11±10.43	36.80±18.07
T3	12.11±5.31*	14.30±3.80	T1	27.56±10.57	37.40±18.10
T5	12.68±4.66*	13.87±3.87	T2	25.83±11.19*	37.10±18.17
			T3	25.06±11.82*	35.10±17.79
			T4	23.44±10.53*	36.00±18.34
			T5	25.06±12.12*	36.90±19.03
Ambulation time (seconds)**			Cadence (steps/min)		
T0	5.26±1.65	6.57±2.03	T0	105.34±13.96	109.50±4.33
T1	4.43±0.61*	6.27±1.80	T1	110.97±10.73	105.10±7.31
T2	4.76±1.24	6.62±2.44	T2	109.27±12.96	105.91±8.81
T3	4.22±0.63*	6.31±1.96	T3	111.39±8.66	108.78±6.84
T4	3.98±0.71*	7.24±2.67	T4	113.17±7.20*	109.05±9.24
T5	3.90±0.64*	6.45±1.88	T5	116.06±8.03*	109.14±6.44
Step count (numbers)**			Velocity (cm/sec)**		
T0	8.97±1.98	11.81±3.29	T0	79.37±18.91	65.38±20.10
T1	8.15±1.32	10.88±2.98	T1	88.67±12.46	70.09±21.10
T2	8.63±2.49	11.61±4.26	T2	85.22±18.02	66.56±24.54
T3	7.85±1.48	11.13±3.43	T3	92.15±10.76	69.27±18.23
T4	7.50±1.35*	12.83±4.13	T4	96.09±12.52*	64.08±23.42
T5	7.55±1.41*	11.50±2.78	T5	100.09±13.63*	67.86±17.96

Abbreviation: SD: Standard deviation; *there is a significant difference in post-hoc test, **there is a significant difference between groups, P<0.05.

Supplementary Table 3. The changes of MDS-UPDRSIII across the study.

MDS-UPDRSIII-MMRM	F value	P value		Tn	Real Group		Sham Group	
					Mean ±SD	post-hoc P	Mean ±SD	post-hoc P
Item 2								
Group	11.093	0.001*		T0	1.4 ±0.788		1.7 ±0.483	
Visit	0.116	0.989		T1	1.4 ±0.788	1.000	1.5 ±0.850	1.000
Group*visit	0.572	0.722		T2	1.3 ±0.822	1.000	1.8 ±0.789	1.000
				T3	1.4 ±0.783	1.000	1.8 ±0.919	1.000
				T4	1.3 ±0.810	1.000	1.7 ±0.823	1.000
				T5	1.2 ±0.717	1.000	1.9 ±0.568	1.000
Item 11								
Group	7.438	0.007*		T0	1.8 ±1.072		1.9 ±1.333	
Visit	0.405	0.845		T1	1.6 ±1.237	1.000	1.9 ±1.101	1.000
Group*visit	0.197	0.964		T2	1.4 ±1.080	1.000	2.1 ±1.287	1.000
				T3	1.4 ±0.988	1.000	1.9 ±1.197	1.000
				T4	1.3 ±1.020	1.000	1.8 ±1.229	1.000
				T5	1.3 ±0.964	1.000	1.9 ±1.197	1.000

Tn: test number; Post-hoc: comparing with the T0. Abbreviation: MMRM: mixed effect model repeated measures. *P<0.05, SD: Standard deviation.

Supplementary Table 4. The values of candence and stride length assessments across the study.

Tn	Real Group Mean ±SD	Sham Group Mean ±SD	MMRM	P value	post-hoc P value	
Cadence (steps/min)						
T0	105.34 ±13.96	109.50 ±4.33	Group	0.404	Real	Sham
T1	110.97 ±10.73	105.10 ±7.31	Visit	0.009*	0.241	1.000
T2	109.27 ±12.96	105.91 ±8.81	Group*visit	0.020*	1.000	1.000
T3	111.39 ±8.66	108.78 ±6.84			0.178	1.000
T4	113.17 ±7.20*	109.05 ±9.24			0.021*	1.000
T5	116.06 ±8.03*	109.14 ±6.44			0.000*	1.000
Stride length (meters)						
T0	0.61 ±0.13	0.48 ±0.15	Group	0.144	Real	Sham
T1	0.65 ±0.09	0.53 ±0.18	Visit	0.262	1.000	1.000
T2	0.64 ±0.15	0.53 ±0.21	Group*visit	0.047*	1.000	1.000
T3	0.68 ±0.11	0.50 ±0.15			0.997	1.000
T4	0.71 ±0.10	0.46 ±0.16			0.166	1.000
T5	0.71 ±0.13	0.48 ±0.14			0.157	1.000

Tn: test number; Post-hoc: comparing with the T0. Abbreviation: MMRM: mixed effect model repeated measures. *P<0.05, SD: Standard deviation.
